# Surgical repair of a pseudocoarctation with cervical aortic arch complicated by multiple aneurysms of the aorta: a case report

**DOI:** 10.11604/pamj.2017.26.236.11800

**Published:** 2017-04-25

**Authors:** Said Makani, Julia Mitchell, Olivier Metton, Sylvie Di Filippo, Roland Henaine, Jean Ninet

**Affiliations:** 1 Department of Cardiovascular and Congenital Surgery, Louis Pradel Hospital, Bron, Lyon, France; 2 Department of Paediatric and Congenital Cardiology, Louis Pradel Hospital, Bron, Lyon, France

**Keywords:** Pseudocoarctation, cervical aortic arch, aneurysm, arch surgery

## Abstract

Aortic pseudocoarctation is a rare congenital anomaly characterized by elongation and deformity of the aortic arch and is known to be associated with aneurysmal formation. Several studies unite to say it leads to a surgical sanction as soon as symptomatic or associated with aneurysms of the aortic arch. Our patient is a 12 years old boy, followed since birth for a little tight pseudocoarctation with a cervical aortic arch and transverse aortic arch hypoplasia. Close clinical and paraclinical monitoring including angioscans, showed the gradual enlargement of the superior mediastinum, in relation with the appearance of three aneurysms of the aortic arch. The intervention, performed by sternotomy, has consisted of the resection of the aneurysmal area and the interposition of a Dacron tube to repair the aortic arch and the reimplantation of the left subclavian artery into the left carotid artery. The postoperative course was uneventful. Management of pseudocoarctation associated with cervical aortic arch and aneurysms remains surgical. Close monitoring of patients with pseudocorctation, seems to be essential to avoid fatal complications such as aneurysmal rupture.

## Introduction

Pseudocoarctation is a rare congenital anomaly defined as kinking of the aorta with normal blood flow dynamics [1], characterized by elongation and deformation of the aortic arch similar to classic coarctation, but in which no obstruction is demonstrable neither collateral circulation [2]. According to several studies, surgical treatment should be the choice for all the symptomatic patients and or those with associated aneurysm formation [3]. We report an uncommon case of pseudocoarctation associated with cervical aortic arch and 3 aneurysmal formations of the aorta, and we focus on the interest of close monitoring of these patients to avoid complications such as aneurysmal rupture.

## Patient and observation

**Figure 1:**
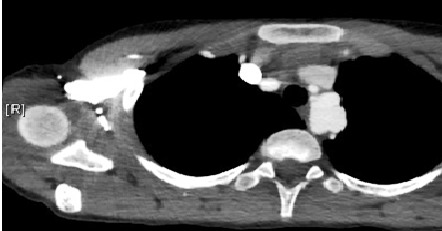
Angioscan showing the saccular aneurysms

**Figure 2:**
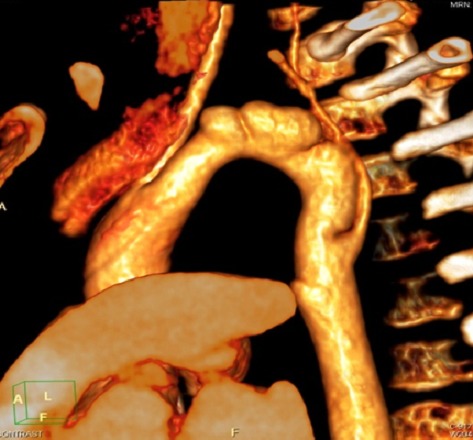
3D reconstruction showing the cervical aortic arch associated to 3 aneurysms of the aortic arch

**Figure 3:**
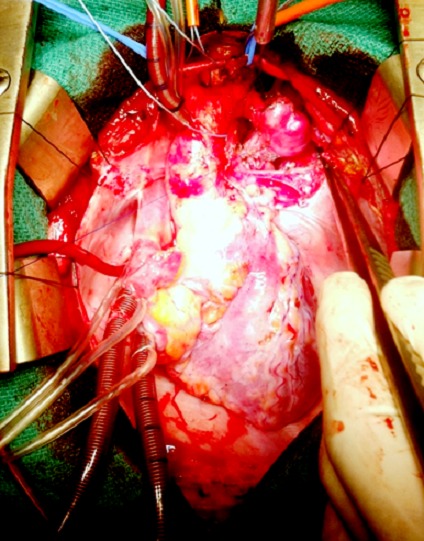
Operative vue of the saccular aneurysms

Our patient is a 12 years old boy, diagnosed with pseudocoarctation in the early childhood, first detected due to abnormal mass seen on chest radiograph. A CT scan has confirmed the diagnosis. During the follow-up, including physical exams, chest radiographs and annual CT scan, we found an enlargement of the mediastinal mass and an obvious protrusion of the aortic knuckle with a cardiothoracic ratio at 45%. In physical examination, we noted a persistent arterial hypertension; with a blood pressure gradient between upper and lower extremities of 36 mmHg. The transthoracic echocardiography showed no left ventricle hypertrophy, no aortic stenosis or insufficiency, and the aortic valve was tricuspid. There was no other congenital cardiac abnormality. A chest CT scan was performed in June 2013; at the age of 11years and showed a relative hypoplasia of the proximal segment of the aortic arch at 10 mm, a cervical aortic arch, three transverse aortic aneurysms (Figure 1) between the left carotid artery and a low implanted left subclavian artery, with a kinking of the aortic isthmus (Figure 2). There was no pleural or cardiac suffusion. We have eliminated the presence of any other aneurysmal dilatation (renal or digestive arteries). A cerebral RMI has eliminated cerebral arteries aneurysm as well. Even if the patient was totally asymptomatic, the persistence of a relative hypertension and the potential risk of rupture made us decide a surgical sanction. The operation was performed through a median sternotomy, we proceed to an extended mobilization of the ascending aorta, the brachio-cephalic artery (BCAT) and the left carotid artery. We desiccate the distal aortic arch, the descending aorta and the left subclavian artery which arises very low and has a parallel path along the aorta. We confirmed the cervical aortic arch and we noted 3 saccular aneurysms developed on the posterior and lateral wall of the aortic arch. The wall was extremely thin (Figure 3). Surgery was performed under cardio pulmonary bypass between a cannula into the ascending aorta, at the foot of BCAT and two venous cannulas, without circulatory arrest. Cooling to 26 °C passage to selective cerebral circulation by pushing the aortic cannula into the BCAT and 1/3 CPB rate. We controlled and temporary ligatured, the BCAT around the cannula, the left carotid artery and the subclavian artery. After cross clamping of the aorta, we put a crystalloid anterograde cardioplegia. We clamped the descending thoracic aorta and cut the distal aortic arch, we noted that the aneurysm wall was extremely thin nearly to rupture; we made an end-to-end anastomosis between a 14 mm Dacron tube and the descending thoracic aorta. To set up supra aortic trunks, we cut the tube in pallet. Finally we made an end-to-side anastomosis between the left subclavian artery and the left side face of the left internal carotid artery. During the operation we noticed the total absence of collateral circulation, the aortic arch was reaching the left clavicle and there was no aortic lumen stenosis.

## Discussion

Pseudocoarctation of the aorta has been described in the literature as a “benign” entity warranting no specific therapy [1]. It seems that its embryologic cause is a failure of compression of the third through the tenth segments of the dorsal aortic roots and the fourth arch segment [2]. Many abnormalities associated with pseudocoarctation were reported such as bicuspid aortic valve, aortic stenosis, ventricular septal defect, atrial septal defect, patent ductus arteriosus, sinus of Valsalva aneurysm, and transposition of the great arteries [2, 3]. The post-operative course was uneventful and our patient was discharged nine days after surgery without any complication. At 2 years follow-up, the patient is well recovering, the control by CT scan is satisfactory and the antihypertensive treatment is already decreased, however we prohibited traumatic sports. Criteria that have been used for diagnosing pseudocoarctation include abnormal chest radiograph, a pressure gradient more than 25 mm Hg between upper and lower extremities, and no evidence of increased collateral circulation. The CT scan provides a definitive diagnosis. In the literature, few report cases of pseudocoarctation of the aorta associated to aortic aneurysm are available [3], the development of an aneurysmal formation is probably due to turbulences in the post-stenotic area. Our patient developed 3 saccular aneurysms during the follow-up. A cervical aortic arch develops most likely from the persistence of the third branchial arch and is complicated by aneurysm in 20% of cases [4]. Cervical aortic arch may be symptomatic; and can be revealed by chest pain [5], oppression in the neck and dizziness, but are usually asymptomatic cases [6]. Recent researches suggest that a cervical aortic arch is associated with deletions in chromosome 22q11, and hence these lesions could be included in the spectrum of defects known as catch 22. This syndrome, described by Wilson and colleagues in 1993, is characterized by cardiac defects, facial dysmorphic, thymic hypoplasia, cleft palate, hypocalcaemia, and a deletion in chromosome 22 [6, 7]. For our patient the decision to proceed to the intervention was made on the combination of cervical aortic arch, pseudocoarctation and the presence of saccular aneurysms, even if the patient was asymptomatic. Unlikely many cases in the literature [8] we made median sternotomy in order to allow us to enlarge the aortic arch and to remove the aneurysmal portion. Our principal teaching point once the diagnosis of pseudocoarctation of the aorta is made is these patients must remain under a program of annual surveillance to identify the possible formation of aneurysm, in order to prevent lethal complication as rupture.

## Conclusion

The association of pseudocoarctation and cervical aortic arch complicated by aneurysms is extremely rare; the treatment is surgical, even for asymptomatic patients, because of the risk of rupture. The close follow-up is mandatory once the diagnosis is done, clinically, with echography and CT scans, to avoid fatal complications.
